# Machine-learning model to predict the tacrolimus concentration and suggest optimal dose in liver transplantation recipients: a multicenter retrospective cohort study

**DOI:** 10.1038/s41598-024-71032-y

**Published:** 2024-08-28

**Authors:** Soo Bin Yoon, Jeong-Moo Lee, Chul-Woo Jung, Kyung-Suk Suh, Kwang-Woong Lee, Nam-Joon Yi, Suk Kyun Hong, YoungRok Choi, Su young Hong, Hyung-Chul Lee

**Affiliations:** 1 Department of Anesthesiology and Pain Medicine, Seoul National University College of Medicine, Seoul National University Hospital, 101 Daehak-ro, Jongno-gu, Seoul, 03080 Republic of Korea; 2 Department of Surgery, Seoul National University College of Medicine, Seoul National University Hospital, Seoul, Republic of Korea

**Keywords:** Tacrolimus concentration, Liver transplantation, Machine-learning algorithm, Long short-term memory, Gradient-boosted regression tree, Computational biology and bioinformatics, Mathematics and computing, Hepatology

## Abstract

Titrating tacrolimus concentration in liver transplantation recipients remains a challenge in the early post-transplant period. This multicenter retrospective cohort study aimed to develop and validate a machine-learning algorithm to predict tacrolimus concentration. Data from 443 patients undergoing liver transplantation between 2017 and 2020 at an academic hospital in South Korea were collected to train machine-learning models. Long short-term memory (LSTM) and gradient-boosted regression tree (GBRT) models were developed using time-series doses and concentrations of tacrolimus with covariates of age, sex, weight, height, liver enzymes, total bilirubin, international normalized ratio, albumin, serum creatinine, and hematocrit. We conducted performance comparisons with linear regression and populational pharmacokinetic models, followed by external validation using the eICU Collaborative Research Database collected in the United States between 2014 and 2015. In the external validation, the LSTM outperformed the GBRT, linear regression, and populational pharmacokinetic models with median performance error (8.8%, 25.3%, 13.9%, and − 11.4%, respectively; *P* < 0.001) and median absolute performance error (22.3%, 33.1%, 26.8%, and 23.4%, respectively; *P* < 0.001). Dosing based on the LSTM model’s suggestions achieved therapeutic concentrations more frequently on the chi-square test (*P* < 0.001). Patients who received doses outside the suggested range were associated with longer ICU stays by an average of 2.5 days (*P* = 0.042). In conclusion, machine learning models showed excellent performance in predicting tacrolimus concentration in liver transplantation recipients and can be useful for concentration titration in these patients.

## Introduction

Tacrolimus is a major immunosuppressive drug in liver transplantation that has been shown to enhance graft survival and reduce recipient mortality compared to other calcineurin inhibitors^1–3^. Nonetheless, the precise dosing of tacrolimus in liver transplantation recipients necessitates meticulous titration due to its narrow therapeutic index^4,5^. Excessive doses of tacrolimus can cause severe complications, such as renal impairment, while insufficient doses can lead to acute rejection^6–8^.

Previous studies have reported large inter-individual variability in the pharmacokinetics of tacrolimus because of multiple reasons, such as genetic polymorphisms of CYP3A5^9–11^. Furthermore, changes in the function of the transplanted liver during the early postoperative phase can trigger considerable intra-individual variability^4,12^. The clinical applicability of conventional population pharmacokinetic (PK) models is limited during the early post-transplant period for liver recipients^9,13,14^. Consequently, an empirical dosing strategy guided by therapeutic drug monitoring is frequently employed in clinical settings. A common approach starts with a dose based on the recipient's body weight, usually 0.1 to 0.15 mg/kg/day, and is adjusted using the target-to-current trough concentration ratio or CYP3A5 genotypes^15,16^. However, these methods are often unsatisfactory in terms of safety and efficiency^17^.

Recent advances in machine-learning techniques have enabled the development of accurate prediction models in various medical fields^18^. In our previous work, we developed a machine-learning model that could predict hypnotic levels during propofol infusion in patients undergoing surgery using the time-series dosing data. Our study demonstrated the superior performance of the machine-learning model over the conventional population PK-pharmacodynamic model^19^. Traditional PK models primarily focus on understandable covariates and their influence on drug concentration, whereas machine-learning models enhance performance by fully exploiting the available data^20^. Such a data-driven approach can be a more pragmatic technique for the drugs with considerable inter- and intra-individual variabilities in the population PK model.

Therefore, this study aimed to develop a machine-learning model to predict tacrolimus concentration in liver transplantation recipients. We hypothesized that a machine-learning model could be developed to predict tacrolimus concentrations in liver transplantation recipients with higher accuracy than that of conventional PK models or linear regression (LR)-based estimations. Furthermore, to transparently evaluate the model’s performance, we conducted external validation using an open dataset, the eICU Collaborative Research Database (eICU-CRD), featuring data collected from geographically and ethnically different cohorts during different periods^21^.

## Results

Overall, 6264 tacrolimus samples were collected up to 14 days post-liver transplantation from 443 patients who underwent liver transplantation at the Seoul National University Hospital (Supplementary Fig. S1). Among this group, 355 (80%) patients were randomly selected to train the model, and the remaining 88 (20%) were used for the test data for internal validation (Table 1). All patients received mycophenolate mofetil and steroids in combination with tacrolimus for immunosuppression.

**Table 1:** General characteristics of the patients in the training and testing groups.

	Total Dataset (n = 443)	Training Dataset (n = 355)	Test Dataset (n = 88)	*P*-value
Sample number (%)	6264 (100%)	5019 (80.1%)	1245 (19.9%)	
Age (years)	55.6 (11.4)	55.4 (11.7)	56.5 (10.2)	0.380
Sex (Male / Female)	306 / 137	245 / 110	61 / 27	0.042
Height (cm)	165.1 (8.0)	165.2 (8.0)	164.5 (8.0)	0.483
Weight (kg)	64.5 (14.7)	64.3 (14.8)	65.1 (14.4)	0.684
Covariates				
AST (IU/L)	52.9 (51.6)	53.7 (52.4)	49.9 (48.6)	0.520
ALT (IU/L)	37.8 (51.0)	39.1 (54.9)	32.9 (31.1)	0.163
Total bilirubin (mg/dlL)	6.0 (10.0)	6.3 (10.3)	5.1 (8.5)	0.264
INR	1.6 (1.7)	1.6 (1.9)	1.4 (0.7)	0.083
Serum albumin (g/dL)	3.3 (0.7)	3.3 (0.6)	3.3 (0.7)	0.671
Serum creatinine (mg/dL)	1.2 (1.1)	1.2 (1.2)	1.1 (1.0)	0.438
Hematocrit (%)	31.9 (6.3)	31.8 (6.3)	32.5 (6.2)	0.328
MELD scores	15.7 (9.3)	15.9 (9.5)	14.7 (8.8)	0.224
Donor (Living/Deceased)	380 / 63	301 / 54	79 / 9	0.426
Indications				
Viral hepatitis B and C	232 (52.5)	187 (52.5)	45 (51.7)	0.996
Neoplasm	102 (23.0)	82 (23.2)	20 (22.5)	
Alcoholic cirrhosis	52 (11.7)	41 (11.6)	11 (12.4)	
Autoimmune disease	31 (7.0)	24 (6.8)	7 (7.9)	
Others	26 (5.9)	21 (5.9)	5 (5.6)	
Tacrolimus				
Daily average dose (mg/day)	3.3 (1.6)	3.5 (1.7)	3.3 (1.6)	0.330
Mean serum concentration (ng/mL)	5.7 (1.2)	5.7 (1.4)	5.6 (1.2)	0.704

Data are expressed as means (standard deviations) or number (percentages).

AST, aspartate aminotransferasel; ALT, alanine transferase; INR, International Normalized Ratio; MELD, Model for End-Stage Liver Disease.

**Fig. 1:**
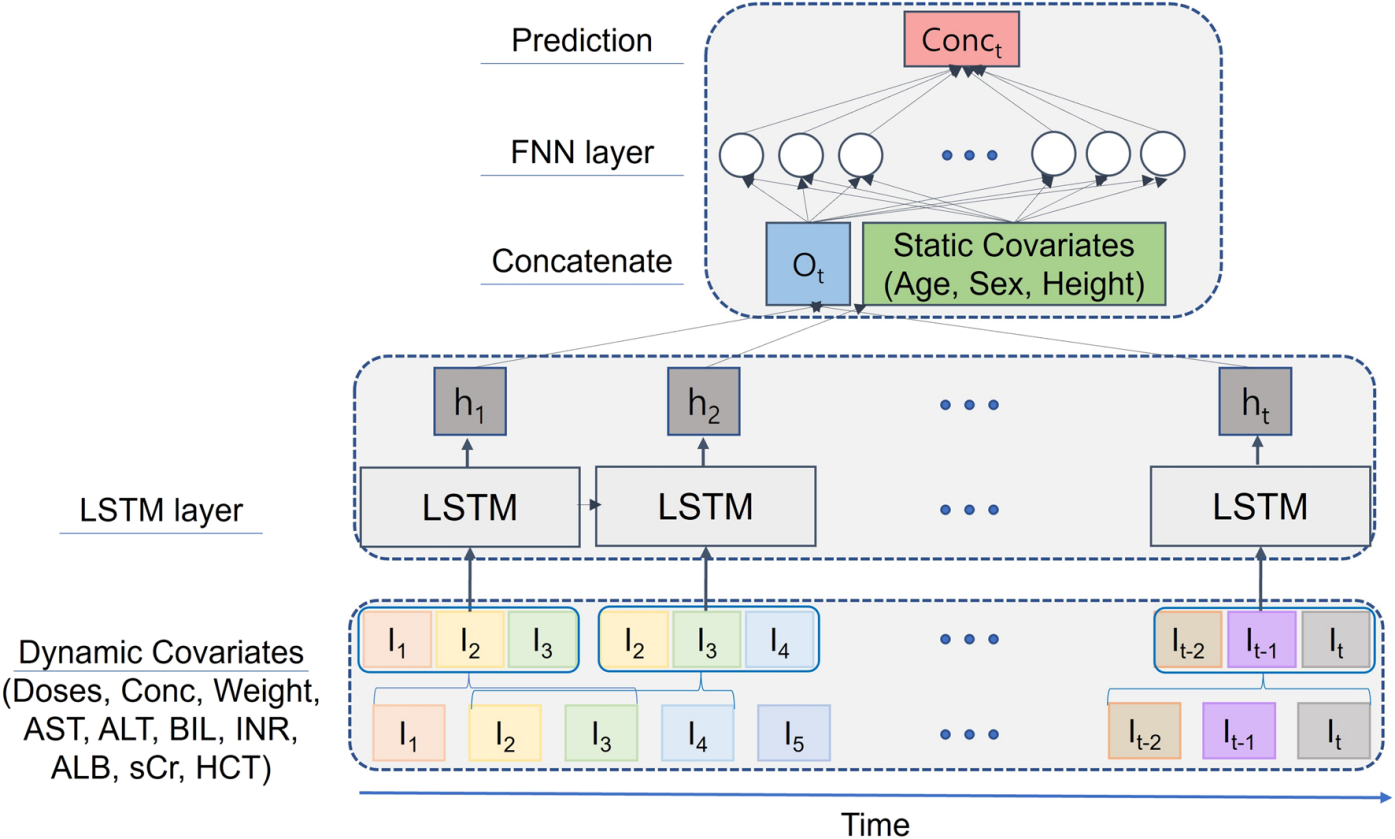
Structure of the machine-learning model. The input data of the LSTM layer were the doses of tacrolimus, measured tacrolimus concentration, and dynamic covariates (weight, AST, ALT, total bilirubin, INR, albumin, serum creatinine, and hematocrit) for 3 days. The output of the LSTM model, O_t_, was concatenated with static covariates (age, sex, and height) and subsequently passed through the FNN layer. The predicting variable was the tacrolimus concentration of the next day (Conc_t_). The solid arrow presents the progress of learning. AST, aspartate aminotransferase; ALT, alanine transferase; BIL, total bilirubin; INR, international normalzed ratio; ALB, serum albumin; sCr, serum creatinine; HCT, hematocrit; FNN, feed-forward neural network; LSTM, long short-term memory.

Figure 1 shows the structure of the model. The best performance was achieved with the following variables: two times daily tacrolimus doses, whole blood tacrolimus concentration, weight, serum AST, and creatinine levels. The details of the feature selection and hyperparameter optimization are provided in Supplementary Table S1 and S2. After a grid search for hyperparameter optimization, the combination of 3 days of data as input, 16 nodes in the LSTM, and 32 nodes in the fully connected layer showed the least validation error. Additionally, the validation errors of the models based on the input features and hyperparameters are provided in Supplementary Table S1 and S2.

The effect of each input feature was illustrated in the SHAP summary plot (Supplementary Fig. S2). Specifically, the increase in previously measured tacrolimus concentration, administered tacrolimus dose, serum AST, and age were associated with a higher level of next tacrolimus concentration.

Both machine-learning models outperformed the conventional LR and the population PK models (Table 2). Specifically, the LSTM exhibited the best predictive performance among the models. The GBRT model was also superior to the PK model regarding the MAE (1.2% vs. 1.4%, *P* < 0.001) and MDAPE (18.5% vs. 19.0%, *P* < 0.001), whereas the RMSE did not significantly differ (1.8 vs. 1.7 ng/mL, *P* = 0.144). Supplementary Fig. S3 illustrates the correlation between the observed tacrolimus blood concentration and the predicted blood concentration from the models.

**Table 2:** Comparison of prediction performance in the internal and external validations.

	Internal validation (n = 88)				External validation (n = 106)			
	LSTM	GBRT	Linear regression	Population PK	LSTM	GBRT	Linear regression	Population PK
	With concentration measurements							
RMSE (ng/mL)	1.7 (0.1)*	1.8 (0.1)*	1.9 (0.1)*	1.7 (0.8) *	1.7 (0.0)	2.2 (0.1)	2.0 (0.0)	2.3 (0.9)
MAE (ng/L)	1.3 (0.0)	1.2 (0.0)	1.3 (0.0)	1.4 (0.7)	1.5 (0.0)	1.9 (0.1)	1.6 (0.0)	1.8 (0.6)
MDPE (%)	− 0.9 (2.2)	6.0 (1.0)	5.3 (1.4)	3.1 (17.1)	8.8 (2.8)	25.3 (2.7)	13.9 (0.9)	− 11.4 (10.3)
MDAPE (%)	16.7 (1.0)	18.5 (0.7)	19.3 (0.8)	19.0 (9.4)	22.3 (1.8)	33.1 (2.5)	26.8 (0.5)	23.4 (10.0)
	Without concentration measurements							
RMSE (ng/mL)	2.3 (0.1)	2.3 (0.1)	2.9 (0.2)	3.7 (1.7)	2.8 (1.6)	3.4 (0.3)	3.4 (0.1)	3.7 (2.0)
MAE (ng/L)	1.8 (0.1)	1.6 (0.0)	2.2 (0.1)	3.3 (1.5)	2.7 (1.6)	2.9 (0.3)	2.8 (0.1)	3.4 (2.0)
MDPE (%)	1.7 (6.9)	8.0 (2.3)	3.6 (2.9)	− 37.6 (34.5)	46.3 (73.6)	51.0 (6.2)	28.0 (3.1)	− 3.3 (54.5)
MDAPE (%)	25.3 (1.8)	25.6 (1.6)	32.2 (2.0)	48.4 (21.5)	57.7 (65.6)	58.4 (47.5)	53.8 (1.2)	47.7 (28.6)

Data are presented as means (standard deviations). All *P* < 0.001 after 10 random trials, except for comparing RMSE in the internal validation* (*P* = 0.144).

GBRT, gradient boosted regression tree; LSTM, long short-term memory; PK, pharmacokinetic; MAE, median absolute error; MDAPE, median absolute performance error; MDPE, median performance error; RMSE, root mean squared error.

In the external validation, the LSTM model trained solely with the Seoul National University Hospital data was applied to the eICU-CRD dataset of 106 liver transplants. Although the overall performance error increased (Table 2), the LSTM model’s performance was maintained in the external validation (RMSE of 1.7 ng/mL, MAE of 1.5 ng/mL, MDPE of 8.8%, and MDAPE of 22.3%). The performances of the GBRT, LR, and PK models were relatively poor in the external validation compared to those of the LSTM model (RMSE, MAE, MDPE, and MDAPE of 2.2 ng/mL, 1.9 ng/mL, 25.3%, and 33.1%, respectively, for the GBRT model; RMSE, MAE, MDPE, and MDAPE of 2.0 ng/mL, 1.6 ng/mL, 13.9%, and 26.8%, respectively, for the LR model; RMSE, MAE, MDPE, and MDAPE of 2.3 ng/mL, 1.8 ng/mL, − 11.4%, and 23.4%, respectively, for the PK model, all *P* < 0.001).

Table 3 compares the predicted tacrolimus concentration with the observed concentration by evaluating the administered dose against the dose suggested by the LSTM model. The results showed that when the patients received the suggested tacrolimus doses predicted by the LSTM model, a significantly high frequency of the actual concentration was within the therapeutic range (*P* < 0.001). The LSTM model identified clinical underdosing or overdosing in 64% of administered doses during the early post-transplant period. ICU stays were longer for patients receiving tacrolimus doses outside the suggestion (193 vs. 250 patients; mean (standard deviation) ICU stay: 8.0 (16.3) vs. 5.5 (5.7) days, *P* = 0.042). Even with suggested doses, concentrations fell outside the target range at rates of 12%, 15%, and 13% for the LSTM, GBRT, and LR models, respectively.

**Table 3:** Number and proportion of patients following the suggested doses of the LSTM model versus achieving the target concentration range.

	Under the target concentration	Within the target concentration	Over the target concentration
Dose over the suggested doses by LSTM	3 (0%)	36 (3%)	31 (3%)
Dose within the suggested doses by LSTM	69 (6%)	116 (10%)	73 (6%)
Dose under the suggested doses by LSTM	690 (61%)	80 (7%)	34 (3%)

Each cell contains the number of cases according to whether the actual dose and tacrolimus concentration were lower, on target, or higher than the dose suggested by the machine-learning model and the target concentration range (8–10 ng/mL), respectively. The observed tacrolimus concentration range significantly differed depending on whether the actual dose was consistent with the suggested dose (*P* < 0.001).

LSTM, long short-term memory.

In clinical outcomes, tacrolimus concentrations exceeding the target range in the early post-transplant period were associated with liver transplant rejection (197 vs. 244 patients; 16% *vs*. 9%, *P* = 0.031). However, exceeding the therapeutic range or high intra-patient variability of tacrolimus were not associated with acute kidney failure or CMV infection.

In the sensitivity analysis, the machine-learning models performed slightly worse when predicting without drug concentration data, although better than the LR and PK models (RMSE, MAE, MDPE, and MDAPE of 2.3 ng/mL, 1.8 ng/mL, 1.7%, and 25.3%, respectively, for the LSTM model; RMSE, MAE, MDPE, and MDAPE of 2.3 ng/mL, 1.6 ng/mL, 8.0%, and 25.6%, respectively, for the GBRT model; RMSE, MAE, MDPE, and MDAPE of 2.9 ng/mL, 2.2 ng/mL, 3.6%, and 32.2%, respectively, for the LR model; RMSE, MAE, MDPE, and MDAPE of 3.7 ng/mL, 3.3 ng/mL, − 37.6%, and 48.4%, respectively, for the LR model, all *P* < 0.001 except for the MDAPE) (Table 2).

## Discussion

This study developed and externally validated machine-learning models to predict tacrolimus concentrations in liver transplantation recipients. Our model showed clinically acceptable performance, superior to the conventional LR and PK models in predicting tacrolimus concentrations during the postoperative period, and it was well maintained in the external validation. Translating tacrolimus concentration predictions into dosage recommendations revealed that deviations from suggested doses were associated with exceeding the target range and prolonged ICU stays.

Several population PK models for predicting tacrolimus concentration have been developed in adult liver transplantation recipients. However, a recent systematic analysis found that these models exhibit inadequate accuracy in external validation^22^. Only 37% of the 16 models reviewed had an acceptable level of accuracy with an MDPE of < 20%, and all 16 models demonstrated poor precision, as indicated by an MDAPE of > 30%^22–24^. These poor outcomes can be attributed to the complex and non-linear kinetics of tacrolimus in liver transplantation recipients. The drug kinetics can be influenced by several factors, such as varying bioavailability^25^, changes in albumin synthesis, erythrocytes where tacrolimus binds^26^, or the distribution and elimination process following biliary complications^27^. Additionally, drug clearance in transplantation recipients is time-dependent since the metabolic function of the liver improves during the early post-transplant stage^28^.

Therefore, to address these complexities, we used a data-driven approach and machine-learning algorithms that could capture the time-dependent non-linear relationship between drug doses and the effect, as we demonstrated in a previous study^22^. Our LSTM-based model showed superior predictive performance in external validation, with an MAE, MDPE, and MDAPE of 1.5 ng/mL, 8.8%, and 22.3%, respectively. These metrics fall within the preset criteria of population PK models for external validation (MDPE ≤  ± 20% and MDAPE ≤  ± 30%)^22–24^. Compared to a previous support vector regression model with an MAE of 2.3 ng/mL our model achieved a 10% reduction in MAE within the target range of tacrolimus (8–10 ng/mL)^29^. These results demonstrate potential for generalizability in predicting tacrolimus concentration in liver transplantation recipients.

Few studies have implemented model-guided dosing algorithms in clinical settings due to the small clinical population and predictive model inaccuracies^30^. Our results showed that patients administered the suggested tacrolimus doses predicted by the LSTM model experienced a considerably higher frequencies of actual concentrations within the therapeutic range. In addition, patients who received doses outside the suggested range were associated with longer ICU stays by an average of 2.5 days (*P* = 0.042). These results align with previous studies demonstrating that personalized, dynamic tacrolimus dosing over time also showed shorter median hospital stays compared to conventional dosing (10 vs. 15 days)^31^. Our model-guided dosing algorithm has the potential to improve patient clinical outcomes when employed during the early post-transplant period.

The small positive bias of the LSTM model in the external validation may be attributed to racial differences in bioavailability. Lu et al. reported that Asians have higher bioavailability than non-Asians^32^. This discrepancy in bioavailability could result in over-prediction of drug concentration when applying models developed for Asians to other racial datasets. However, variations in measurement methods or factors, including residual noise, could also contribute to these differences. Therefore, further analysis is necessary for validation.

Among the various combinations of clinical covariates reflecting overall graft function (ALT, AST, total bilirubin, and INR)^33^, our model incorporating AST demonstrated better performance. AST and ALT levels are sensitive markers of hepatocellular injury within the first 7 days post-transplantation period, rapidly reflecting graft function^33,34^. In contrast, total bilirubin and INR levels during the first 6 days post-transplantation could be influenced more by the recipient's pre-transplant status than by the new graft function^33,34^. This distinction may explain the superior performance of our model incorporating AST over other covariates during rapid changes of liver function in the early post-transplant period.

While we propose using machine learning-assisted concentration prediction and dose adjustment for tacrolimus, therapeutic drug monitoring remains essential. The reduced predictive capability of the model without concentration data highlights the necessity for therapeutic drug monitoring. In addition, the LSTM model misidentified the administered doses as correct doses in 12% of test datasets. Suggesting the median of the possible dose combinations expected to fall within the target range could reduce incorrect dose suggestions, but requires laboratory confirmaiton. The benefits and feasibility of the LSTM-assisted approach in suggesting tacrolimus doses, alongside therapeutic drug monitoring, warrants further confirmation in prospective studies.

Our study had some limitations. First, although our model’s performance was externally validated in different races and locations, this is a retrospective study, and bias may exist. For example, the clinician’s aim to maintain a proper tacrolimus concentration resulted in an imbalanced data distribution with limited data outside of the target range and poor predictive performance^35^. Therefore, additional data beyond the clinical range can improve our model’s accuracy. Second, although we replaced the missing values using multiple imputations, our model still requires several laboratory tests, such as those involving serum ALB, creatinine, ALT, AST, and HCT. These results may not be available daily for three consecutive days at some centers, either due to protocol differences or resource limitations, particularly in developing countries. Third, additional covariates, such as the genotype of metabolic enzymes, might affect the tacrolimus dose–concentration relationship. However, adding these covariates to the model remains difficult in most clinical settings. Fourth, our model for twice-daily dosing in the early post-operative period has limited applicability for patients rapidly converting to once-daily dosing^36^. Future studies on predictive models for once-daily dosing could address this limitation.

In conclusion, we developed machine-learning models that predict tacrolimus concentrations in liver transplantation recipients. Our LSTM model demonstrated excellent performance in external validation. Dosing based on the model’s suggestions were resulted in concentrations within the therapeutic range in more cases. Patients who received doses outside the suggested range were associated with longer ICU stays. Therefore, this approach can be useful for accurately predicting tacrolimus concentrations and suggesting appropriate doses for patients undergoing liver transplantation to improve clinical outcomes.

This underscores the potential of machine learning algorithms for tacrolimus concentration prediction and dosage suggestions to enhance patient outcomes.

## Patients and methods

### Study approval

This study was conducted in accordance with the tenets of the Declaration of Helsinki. The Institutional Review Board of Seoul National University Hospital approved the study proposal (approval number: H-2007-083-1141) and waived the requirement for written informed consent due to the retrospective study design. After obtaining approval, we retrospectively collected data from patients who underwent liver transplantation between January 2017 and October 2020. Patients aged < 15 years or those without any record of tacrolimus concentrations were excluded. We followed the recommendations from the article “STROCSS 2021: Strengthening the Reporting of Cohort, Cross-sectional and Case–control Studies in Surgery”^37^.

### Data collection

The two times daily doses of tacrolimus up to 14 days postoperatively and whole blood tacrolimus concentration measured by chemiluminescence immunoassay were collected from electronic medical records at the Seoul National University Hospital for model training and internal validation. Additionally, the patient’s age, sex, height, weight, Model for End-Stage Liver Disease (MELD) score, type of donor, indication for transplant, other immunosuppresants were recorded. Blood test results for alanine aminotransferase (ALT), aspartate aminotransferase (AST), total bilirubin, International Normalized Ratio (INR), serum albumin, serum creatinine, hematocrit were collected daily^38^.

During the study period, the patients were administered an oral dosage of tacrolimus two times daily from the 1st day after liver transplantation. Doses were empirically decided by the attending intensivists based on the patient’s weight, laboratory results related to liver and renal functions, and the whole blood tacrolimus concentration measured before taking the morning dose of the medication. Dose control and drug concentration monitoring were repeated until the tacrolimus concentration reached a steady-state concentration in the target range between 8 and 10 ng/mL.

### Model development

A machine-learning model was developed to predict the next whole tacrolimus concentration test results based on the history of oral tacrolimus doses, measured whole blood tacrolimus concentrations, time-dependent covariates (weight, ALT, AST, total bilirubin, INR, serum albumin, serum creatinine, hematocrit) of previous *n* days, and time-independent covariates (age, sex, and height). The dataset comprised the variables for *n* + *1* consecutive days, the first *n* days for inputs, and the last day for output. Furthermore, the missing values were imputed using multiple imputations. The concentrations and doses of the tacrolimus before the first administration were substituted with zeros.

A long short-term memory (LSTM) model was developed using the input nodes of the tacrolimus dose, measured tacrolimus concentration, and time-dependent covariates. The LSTM outputs were concatenated with time-independent covariates and entered into the fully connected layer. These structures were inspired by Lee et al.’s study^19^.

Gradient-boosted regression tree (GBRT) and LR models have also been developed for comparison. These models received the same inputs as the final LSTM model based on data from the previous *n* days. GBRT hyperparameters, such as the number of estimators and maximal depth, were optimized using a similar method.

We employed a one-compartment PK model with first-order absorption developed for patients in the first 2 weeks post-liver transplantation^39^. The PK parameters were adjusted based on the post-transplant stage and the serum albumin, AST, or hematocrit measurements: apparent clearance (CL/F) of 8.93 and 11.0 L/h for AST ≥ 500 and < 500 U/L, respectively, and apparent volume (V/F) of 328 L between 0 and 3 days post-transplantation period. After 4 days, apparent clearance was set to 25.1 L/h for serum albumin of < 2.5 g/dL or hematocrit of < 28% and 17.1 L/h otherwise with an apparent volume of 568 L.

Once the best combination of features and hyperparameters was identified, multiple random sampling was performed to evaluate the models’ internal and external validation performance.

Training and validation of the models were performed by the author’s program written in Python (version 3.10.5) using the Keras library (version 2.10.0).

We compared the accuracy of the models with all combinations of the abovementioned variables for feature selection. Among the various combinations, the one with the highest performance and fewer variables in the five-fold cross-validation was selected. A grid search was performed to determine the optimal combination of hyperparameters. Possible combinations of the hyperparameters were 8, 16, 32, 64, 128, and 256 for the number of nodes in the LSTM; 8, 16, 32, 64, and 128 for the number of nodes in the fully connected layer; and 2–7 days for the number of days for input.

To enhance the model transparency and reveal the effects of the input features on the next tacrolimus concentration, we applied the Shapley Additive exPlanations (SHAP) algorithm to further visualize the explanation at the feature level using SHAP version 0.39.0 in Python^40^. Briefly, the SHAP summary plot was used to illustrate the strength and the direction of associations between features and tacrolimus concentration.

### Internal validation

Multiple random sample validations were conducted. The samples in the derivation cohorts were classified into training (80%) and test (20%) sets using 10 random seeds. Subsequently, the training of the model was repeated using similar methods to estimate the mean performance and 95% confidence interval^41^. The predictive performance was evaluated based on the root-mean-squared error (RMSE), median absolute error (MAE), median performance error (MDPE), and median absolute performance error (MDAPE). The agreement between the predicted and measured tacrolimus concentrations was evaluated for each model.

### External validation

For external validation, this study analyzed data from the eICU-CRD dataset, which included over 200,000 intensive care unit stays from 208 hospitals in the United States between 2014 and 2015^21^. The “patient unit stay id” of patients whose admission diagnosis was “liver transplantation” was extracted from the “admission dx” table. Patients aged < 15 years were excluded. Whole blood tacrolimus concentration, ALT, AST, total bilirubin, INR, serum albumin, serum creatinine, and hematocrit measurements (labeled as “lab result offset”) were queried from the “lab” table. The tacrolimus doses were retrieved from the “medication” table and aligned with the lab result based on “drug start offset,” “drug stop offset,” and “lab result offset.” Cases were excluded when the route of drug administration was sublingual or intravenous instead of oral. Data on age, sex, height, and weight were obtained from the “patient” table. Data with missing drug doses or concentrations were excluded to ensure consistency with the training dataset. The LSTM, GBRT, and LR models predicted tacrolimus concentrations in this dataset to confirm the external validity of the model performance.

### Dose recommendation

The model suggested tacrolimus doses by first predicting the tacrolimus concentration for all hypothetical doses between the minimum (0.5 mg) and maximum doses (20 mg). The tacrolimus doses predicted to achieve the target concentration range (8–10 ng/mL) were then identified as the suggested doses. A 3 × 3 contingency table was produced by juxtaposing the administered dose against the suggested doses and the actual measured concentration within the therapeutic range. Subsequently, these frequencies were statistically examined using the chi-square test.

We further evaluated whether dose adjustments aligned with the suggested tacrolimus doses were associated with expedited ICU discharges. We compared the duration of ICU stays between patients who received tacrolimus doses within and outside the suggested range.

### Clinical outcome

We investigated whether tacrolimus concentrations outside the target range or high intra-patient variability, defined as a standard deviation of tacrolimus concetnration over 2 ng/ml, significantly impacted prognosis during the first 14 days post-transplant^42^. The clinical outcomes evaluated included transplantation rejection, renal failure, and CMV infection. Transplant rejection was assessed by transplant surgeons based on laboratory findings, biopsy results, and imaging examinations^43^. Acute kidney failure was defined as an increase in serum creatinine by 0.3 mg/dL or more within 48 h or an increase to 1.5 to 1.9 times baseline within the previous 7 days^44^. CMV infection was diagnosed using PCR assays^45^. We used the chi-squared test to analyze the association between tacrolimus concentration and clinical outcomes during the early post-transplant period.

### Sensitivity analysis

Sensitivity analyses were performed to confirm the robustness of the LSTM model. Specifically, we trained the models without any drug concentration results and evaluated their performance.

### Statistical analysis

Formal sample size calculation was not performed because of the inherent nature of retrospective studies. Instead, the study used available data from tertiary hospitals and a large open dataset to develop and test the prediction model. The patient demographics and doses and concentrations of tacrolimus are described as means (± standard deviations) or medians (interquartile ranges), depending on the results of the Shapiro–Wilk test, and the categorical variables are presented numerically (percentages). Continuous variables, such as the doses and concentrations of tacrolimus, age, weight, height, AST, ALT, total bilirubin, INR, serum albumin, serum creatinine, and hematocrit were compared using the Student’s *t*-test or the Mann–Whitney U-test. Categorical variables, such as patient sex, were compared using Pearson’s chi-square test.

Model performance was evaluated using internal test and external validation datasets. The RMSE, MAE, MDPE, and MDAPE were compared using analysis of variance, followed by a *post-hoc* t-test with Bonferroni correction. An MDPE of < 20% or MDAPE of < 30% was determined to be clinically acceptable based on previous studies^22–24^.

Statistical analyses were performed using Python and IBM SPSS for Windows, version 21 (IBM, Armonk, NY, USA), and a significant difference was considered at *P* < 0.05. The code used for the analysis is attached in Supplementary Table S4.

### Supplementary Information

Supplementary Figures.

Supplementary Tables.

## Data Availability

The data that support the findings of this study are available from the corresponding author upon reasonable request.

## Code availability

The code used for the analysis is provided in Supplementary Table S4.
