# Supplementary material for: Machine-learning model to predict the tacrolimus concentration and suggest optimal dose in liver transplantation recipients: a multicenter retrospective cohort study
Source: Sci Rep. 2024 Aug 28;14:19996. doi: 10.1038/s41598-024-71032-y (PMC11358263; doi:10.1038/s41598-024-71032-y)
Supplement: Supplementary file 1 — Supplementary Figures. [file 41598_2024_71032_MOESM1_ESM.docx]

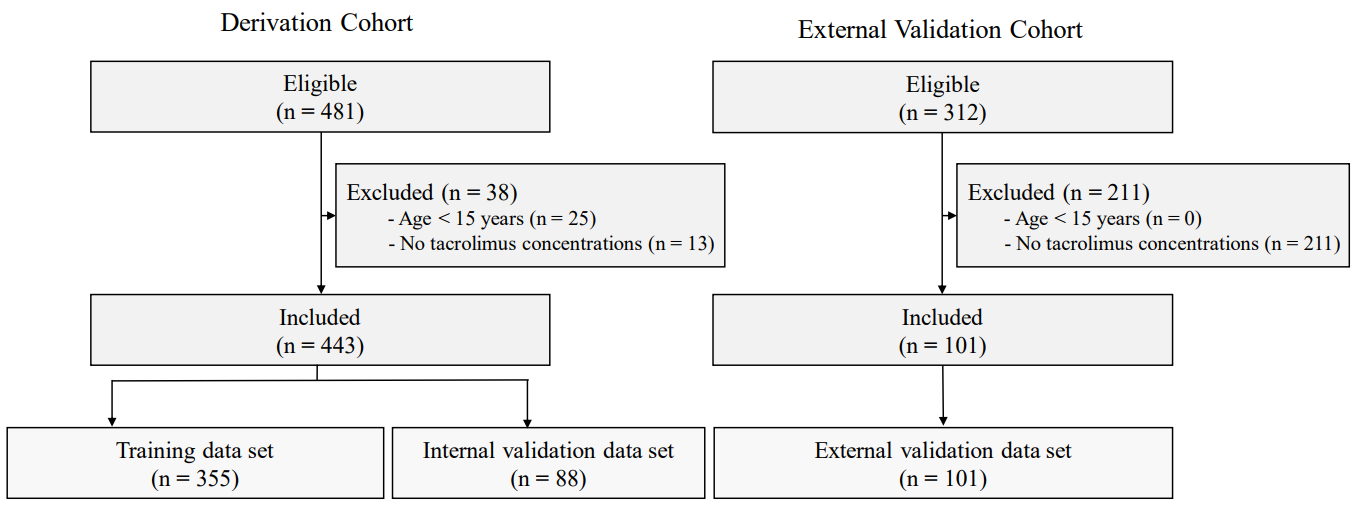


**Supplementary Figure S1.** Retrospective cohort study design and study flow chart.


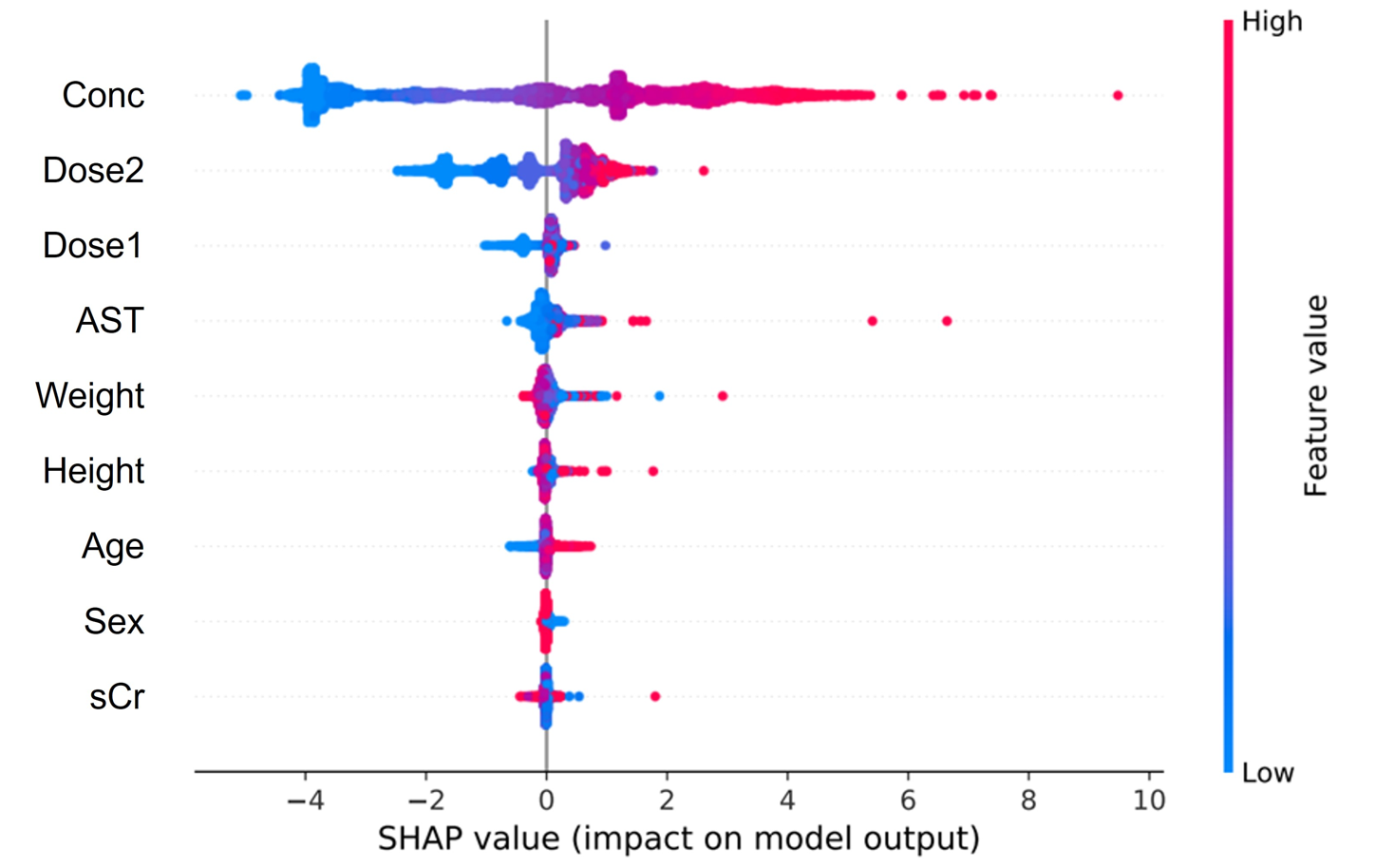


**Supplementary Figure S2.** SHapley Additive exPlanation (SHAP) summary plot illustrates the impact of input features on the tacrolimus concentration. Conc, concentration of previously measured tacrolimus; AST, aspartate aminotransferase; Dose 1, morning dose of tacrolimus; Dose 2, evening dose of tacrolimus; sCr, serum creatinine.


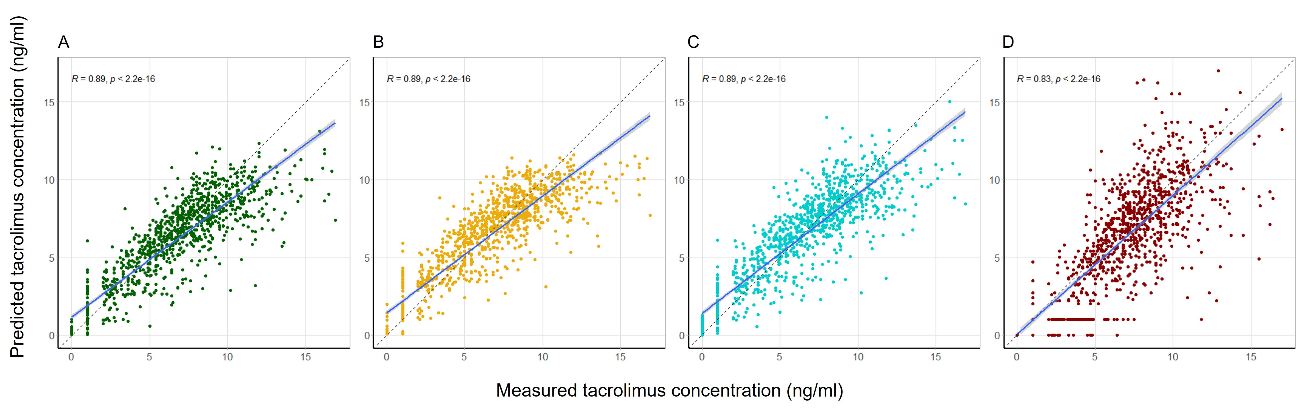


**Supplementary Figure S3.** Predicted versus measured tacrolimus concentration for (A) LSTM, (B) GBRT, (c) LR, and (D) population PK models. The dashed lines represent the line of identity. LSTM, long short-term memory; GBRT, gradient boosted regression tree; LR, linear regression; PK, pharmacokinetic.
