# Supplementary material for: Machine-learning model to predict the tacrolimus concentration and suggest optimal dose in liver transplantation recipients: a multicenter retrospective cohort study
Source: Sci Rep. 2024 Aug 28;14:19996. doi: 10.1038/s41598-024-71032-y (PMC11358263; doi:10.1038/s41598-024-71032-y)
Supplement: Supplementary file 2 — Supplementary Tables. [file 41598_2024_71032_MOESM2_ESM.docx]

**Supplementary Table S1. Feature Selection**

To determine the covariates to be included in the final model, we compared the root-mean-squared error of models with all combinations of time-dependent covariates as follows: body weight, aspartate aminotransferase (AST), alanine aminotransferase (ALT), serum total bilirubin, international normalized ratio (INR), total bilirubin, serum albumin,serum creatinine, and hematocrit. The doses of oral tacrolimus and measured serum concentrations of tacrolimus were consistently included.

| Parameters in the model | RMSE (ng/dL) |
| --- | --- |
| Dose1, Dose2, Conc, Body weight, AST, Creatinine | 1.69 |
| Dose1, Dose2, Conc, Body weight, AST, INR | 1.69 |
| Dose1, Dose2, Conc, Body weight, AST, Hematocrit | 1.7 |
| Dose1, Dose2, Conc, Body weight, AST, Creatinine, Albumin | 1.7 |
| Dose1, Dose2, Conc, Body weight, Creatinine, Total bilirubin, INR | 1.7 |
| Dose1, Dose2, Conc, Body weight, Creatinine, INR | 1.71 |
| Dose1, Dose2, Conc, Body weight, Albumin, Hematocrit, INR | 1.71 |
| Dose1, Dose2, Conc, Body weight, Hematocrit, Total bilirubin, INR | 1.71 |
| Dose1, Dose2, Conc, Body weight, AST, Creatinine, Total bilirubin, INR | 1.71 |
| Dose1, Dose2, Conc, Body weight, Creatinine, Albumin, Hematocrit, Total bilirubin | 1.71 |
| Dose1, Dose2, Conc, Body weight, Creatinine, Albumin, Hematocrit, INR | 1.71 |
| Dose1, Dose2, Conc, Body weight, AST, Creatinine, Albumin, Hematocrit, Total bilirubin, INR | 1.71 |
| Dose1, Dose2, Conc, Body weight | 1.72 |
| Dose1, Dose2, Conc, Body weight, Creatinine, Albumin | 1.72 |
| Dose1, Dose2, Conc, Body weight, Total bilirubin, INR | 1.72 |
| Dose1, Dose2, Conc, Body weight, Albumin, Hematocrit, Total bilirubin | 1.72 |
| Dose1, Dose2, Conc, Body weight, Creatinine, Albumin, Hematocrit, Total bilirubin, INR | 1.72 |
| Dose1, Dose2, Conc, Body weight, Albumin, Hematocrit | 1.73 |
| Dose1, Dose2, Conc, Body weight, Albumin, Total bilirubin | 1.73 |
| Dose1, Dose2, Conc, Body weight, Hematocrit, Total bilirubin | 1.73 |
| Dose1, Dose2, Conc, Body weight, Creatinine, Hematocrit, Total bilirubin | 1.73 |
| Dose1, Dose2, Conc, Body weight, AST, Hematocrit, Total bilirubin, INR | 1.73 |
| Dose1, Dose2, Conc, Body weight, Albumin | 1.74 |
| Dose1, Dose2, Conc, Body weight, Creatinine, Hematocrit | 1.74 |
| Dose1, Dose2, Conc, Body weight, AST, Creatinine, Total bilirubin | 1.74 |
| Dose1, Dose2, Conc, Body weight, Creatinine, Albumin, Hematocrit | 1.74 |
| Dose1, Dose2, Conc, Body weight, AST, Albumin, Hematocrit, Total bilirubin, INR | 1.74 |
| Dose1, Dose2, Conc, Body weight, Hematocrit | 1.75 |
| Dose1, Dose2, Conc, Body weight, Total bilirubin | 1.75 |
| Dose1, Dose2, Conc, Body weight, Albumin, INR | 1.75 |
| Dose1, Dose2, Conc, Body weight, AST, Albumin, Hematocrit | 1.75 |
| Dose1, Dose2, Conc, Body weight, AST, Albumin, Total bilirubin | 1.75 |
| Dose1, Dose2, Conc, Body weight, AST, Total bilirubin, INR | 1.75 |
| Dose1, Dose2, Conc, Body weight, Albumin, Total bilirubin, INR | 1.75 |
| Dose1, Dose2, Conc, Body weight, AST, Albumin, Total bilirubin, INR | 1.75 |
| Dose1, Dose2, Conc, Body weight, Creatinine, Albumin, Total bilirubin, INR | 1.75 |
| Dose1, Dose2, Conc, Body weight, Creatinine, Hematocrit, Total bilirubin, INR | 1.75 |
| Dose1, Dose2, Conc, Body weight, AST, Albumin | 1.76 |
| Dose1, Dose2, Conc, Body weight, Creatinine, Total bilirubin | 1.76 |
| Dose1, Dose2, Conc, Body weight, AST, Hematocrit, Total bilirubin | 1.76 |
| Dose1, Dose2, Conc, Body weight, Creatinine, Albumin, Total bilirubin | 1.76 |
| Dose1, Dose2, Conc, Body weight, AST, Albumin, Hematocrit, Total bilirubin | 1.76 |
| Dose1, Dose2, Conc, Body weight, AST | 1.77 |
| Dose1, Dose2, Conc, Body weight, Hematocrit, INR | 1.77 |
| Dose1, Dose2, Conc, Body weight, AST, Albumin, INR | 1.77 |
| Dose1, Dose2, Conc, Body weight, AST, Creatinine, Albumin, Hematocrit | 1.77 |
| Dose1, Dose2, Conc, Body weight, AST, Albumin, Hematocrit, INR | 1.77 |
| Dose1, Dose2, Conc, Body weight, AST, Creatinine, Albumin, Total bilirubin, INR | 1.77 |
| Dose1, Dose2, Conc, Body weight, AST, Creatinine, Hematocrit, Total bilirubin, INR | 1.77 |
| Dose1, Dose2, Conc, Body weight, INR | 1.78 |
| Dose1, Dose2, Conc, Body weight, AST, Total bilirubin | 1.78 |
| Dose1, Dose2, Conc, Body weight, AST, Creatinine, Hematocrit | 1.78 |
| Dose1, Dose2, Conc, Body weight, AST, Creatinine, Hematocrit, Total bilirubin | 1.78 |
| Dose1, Dose2, Conc, Body weight, AST, Creatinine, Hematocrit, INR | 1.78 |
| Dose1, Dose2, Conc, Body weight, Albumin, Hematocrit, Total bilirubin, INR | 1.78 |
| Dose1, Dose2, Conc, Body weight, Creatinine, Hematocrit, INR | 1.79 |
| Dose1, Dose2, Conc, Body weight, AST, Creatinine, Albumin, Total bilirubin | 1.79 |
| Dose1, Dose2, Conc, Body weight, AST, Creatinine, Albumin, Hematocrit, Total bilirubin | 1.79 |
| Dose1, Dose2, Conc, Body weight, AST, Creatinine, Albumin, INR | 1.81 |
| Dose1, Dose2, Conc, Body weight, AST, Creatinine, Albumin, Hematocrit, INR | 1.82 |
| Dose1, Dose2, Conc, Body weight, AST, Hematocrit, INR | 1.84 |
| Dose1, Dose2, Conc, Body weight, Creatinine, Albumin, INR | 1.88 |
| Dose1, Dose2, Conc, Body weight, Creatinine | 1.89 |

Dose1, morning dose of tacrolimus; Dose2, evening dose of tacrolimus; Conc, concentration of previously measured tacrolimus; RMSE, root-mean-squared error.

**Supplementary Table S2. Hyperparameter optimization**

Hyperparameter optimization with grid search was performed. The number of nodes in LSTM and FNN were examined for 8, 16, 32, 64, and 128.

| The number of LSTM node | The number of FNN node | RMSE (ng/dL) |
| --- | --- | --- |
| 8 | 8 | 1.807 |
| 8 | 16 | 1.771 |
| 8 | 32 | 1.748 |
| 8 | 64 | 1.749 |
| 8 | 128 | 1.764 |
| 16 | 8 | 1.787 |
| 16 | 16 | 1.759 |
| 16 | 32 | 1.724 |
| 16 | 64 | 1.730 |
| 16 | 128 | 1.746 |
| 32 | 8 | 1.797 |
| 32 | 16 | 1.751 |
| 32 | 32 | 1.748 |
| 32 | 64 | 1.740 |
| 32 | 128 | 1.735 |
| 64 | 8 | 1.756 |
| 64 | 16 | 1.745 |
| 64 | 32 | 1.742 |
| 64 | 64 | 1.750 |
| 64 | 128 | 1.738 |
| 128 | 8 | 1.856 |
| 128 | 16 | 1.758 |
| 128 | 32 | 1.739 |
| 128 | 64 | 1.780 |
| 128 | 128 | 1.736 |
| 256 | 8 | 1.775 |
| 256 | 16 | 1.729 |
| 256 | 32 | 1.785 |
| 256 | 64 | 1.743 |
| 256 | 128 | 1.733 |

FNN, feed-forward neural network; LSTM, long short-term memory; RMSE, root-mean-squared error.

**Supplementary Table S3. Number and proportion of patients following the suggested doses of the GBRT and LR models versus achieving the target concentration range.**

|  | Under the target concentration | Within the target concentration | Over the target concentration |
| --- | --- | --- | --- |
| Dose over the suggested doses by GBRT | 3 (0%) | 31 (3%) | 42 (4%) |
| Dose within the suggested doses by GBRT | 85 (8%) | 134 (12%) | 75 (7%) |
| Dose under the suggested doses by GBRT | 674 (60%) | 67 (6%) | 21 (2%) |
| Dose over the suggested doses by LR | 9 (1%) | 44 (4%) | 43 (4%) |
| Dose within the suggested doses by LR | 82 (7%) | 119 (11%) | 71 (6%) |
| Dose under the suggested doses by LR | 674 (59%) | 69 (6%) | 24 (2%) |

**Supplementary Table S4. Available Code**

| import numpy as np  import pandas as pd  import os, csv  from sklearn.model_selection import KFold  import statistic  from sklearn.linear_model import LinearRegression  import tensorflow as tf  from tensorflow.keras.models import Model  from tensorflow.keras.layers import Dropout, Input, Dense, concatenate, LSTM  from tensorflow.keras.callbacks import ModelCheckpoint, EarlyStopping  import sklearn.preprocessing as pcs  import xgboost  # os.environ["CUDA_VISIBLE_DEVICES"] = ""  def Linearmodel(Xtrain, Ytrain, Xtest):  xtrain = Xtrain.reshape(Xtrain.shape[0], -1)  xtest = Xtest.reshape(Xtest.shape[0], -1)  reg = LinearRegression().fit(xtrain, Ytrain)  Ypred_Linear = reg.predict(xtest)  return Ypred_Linear  def datagenerator(data, ids, nday, lstmpara, covpara):  X, Y, C, ID, POD = [], [], [], [], []  for id in ids:  temp = data[data['caseid'] == id][lstmpara].values  tempcov = data[data['caseid'] == id][covpara].values  tempy = data[data['caseid'] == id]['conc'].values  temppod = data[data['caseid'] == id]['pod'].values  for i in range(nday-1):  temp = np.vstack([temp[0], temp])  tempy = np.insert(tempy, 0, tempy[0])  tempcov = np.vstack([tempcov[0], tempcov])  temppod = np.insert(temppod, 0, temppod[0])  templen = int(temp.shape[0])  for i in range(templen-nday):  x = temp[i:(i+nday)]  y = tempy[i+nday]  cov = tempcov[i+nday-1]  pod = temppod[i+nday]  if np.any(np.isnan(x)) or np.isnan(y) or np.any(np.isnan(cov)):  continue  X.append(x)  Y.append(y)  C.append(cov)  ID.append(id)  POD.append(pod)  X = np.array(X)  Y = np.array(Y)  C = np.array(C)  ID = np.array(ID)  POD = np.array(POD)  return X, C, Y, ID, POD  # -----------------------------------------------  epoch = 10  nday = 3  ntimeseq = nday  LSTM_NODES = 16  FNN_NODES = 32  dropout = 0.1  lstmact = 'relu'  fnnact = 'relu'  num_timeseq = nday  random_seed = 42  lstmpara =['dose1', 'dose2', 'conc', 'wt','ot','gfr']  covpara = ['age','ht','sex']  file = 'dataload_final.csv'  data = pd.read_csv(file, dtype=np.float32)  # -----------------------------------------------  ## data preprocessing  data[data<0] = np.nan  RS = pcs.RobustScaler()  SS = pcs.StandardScaler()  MMS = pcs.MinMaxScaler()  for i in ['ot', 'pt', 'gfr', 'alb', 'hct','inr','tbil']: # age  temp = data[i].values  id = ~np.isnan(temp)  temp = temp[id]  data[i][id] = MMS.fit_transform(temp[:, None])[:, 0]  data['age']=MMS.fit_transform(data['age'].values.reshape(-1, 1))  data['ht']=MMS.fit_transform(data['ht'].values.reshape(-1, 1))  data['wt']=MMS.fit_transform(data['wt'].values.reshape(-1, 1))  ## model path allocation  TRAIN = True  parastr = '_'.join(lstmpara)  weight_path = f"weight/{parastr}.hdf5"  if TRAIN:  if os.path.exists(weight_path):  os.remove(weight_path)  result = []  for trainmask, testmask in KFold(n_splits=5, random_state=random_seed, shuffle=True).split(rcaseids):  rcaseids = np.unique(data['caseid'].values)  testids = rcaseids[testmask]  trainids = rcaseids[trainmask]  #train, test dataset generation  Xtest, Covtest, Ytest, IDtest, PODtest = datagenerator(data, testids, nday, lstmpara, covpara)  Xtrain, Covtrain, Ytrain, IDtrain, PODtrain = datagenerator(data, trainids, nday, lstmpara, covpara)  # Define the input layers  ninput = len(lstmpara)  ncov = len(covpara)  input_seq = Input(shape=(ntimeseq, ninput), name='input_layer')  input_cov = Input(shape=(ncov,), name='input_cov')  lstm = LSTM(LSTM_NODES, activation=lstmact)(input_seq)  output = concatenate([lstm, input_cov])  output = Dense(FNN_NODES, activation=fnnact)(output)  output = Dropout(dropout)(output)  main_output = Dense(1, name='main_output')(output)    if TRAIN:  model = Model(inputs=[input_seq, input_cov], outputs=main_output)  model.compile(loss='mae', optimizer='adam')  hist = model.fit({'input_layer': Xtrain, 'input_cov': Covtrain}, Ytrain, validation_split=0.1,  epochs=epoch, batch_size=16, # sample_weight=train_sw,)  callbacks=[ModelCheckpoint(monitor='val_loss', filepath=weight_path, verbose=1, save_best_only=True),  EarlyStopping(monitor='val_loss', patience=1, verbose=0, mode='auto')])  model = tf.keras.models.load_model(weight_path)  Ypred_lstm = model.predict([Xtest, Covtest]).flatten()  rmse_lstm = np.mean(np.square(Ypred_lstm - Ytest))**0.5  rmse_lstm = np.round(rmse_lstm,2)  Xtrain_xgb = np.hstack([Xtrain.reshape(Xtrain.shape[0], -1), Covtrain])  Xtest_xgb = np.hstack([Xtest.reshape(Xtest.shape[0], -1), Covtest])  minimum = 1e10  for n_estimator in [30, 40, 50]:  for max_depth in [3,4,5]:  xgbmodel = xgboost.XGBRegressor(n_estimators=n_estimator, learning_rate=0.08, gamma=0, subsample=0.75, colsample_bytree=1, max_depth=max_depth)  xgbmodel.fit(Xtrain_xgb, Ytrain)  Ypred_xgb = xgbmodel.predict(Xtest_xgb)  rmse_xgb = np.mean(np.square(Ypred_xgb - Ytest))**0.5  if rmse_xgb < minimum:  bestn = n_estimator  bestdepth = max_depth  minimum = rmse_xgb    xgbmodel = xgboost.XGBRegressor(n_estimators=bestn, learning_rate=0.08, gamma=0, subsample=0.75, colsample_bytree=1, max_depth=bestdepth)  xgbmodel.fit(Xtrain_xgb, Ytrain)  Ypred_xgb = xgbmodel.predict(Xtest_xgb)  Xtrain_lr, Xtest_lr = np.copy(Xtrain_xgb), np.copy(Xtest_xgb)  lrmodel = LinearRegression().fit(Xtrain_lr, Ytrain)  Ypred_lr = lrmodel.predict(Xtest_lr)  for modeltype, Ypred in zip(['lstm','xgb','lr'],[Ypred_lstm, Ypred_xgb,Ypred_lr]):  mdpe, mdape, rmse , mae = statistic.mdpe(testids, IDtest, Ypred, Ytest)  result.append({"datafrom":"snu","para":parastr,"modeltype":modeltype, "itrial":itrial,"para":lstmpara, "mdpe":mdpe,"mdape":mdape,"rmse":rmse,"mae":mae})    break  rstdf = pd.DataFrame(result)  print(rstdf) |
| --- |
